# Flavonoids and Mitochondria: Activation of Cytoprotective Pathways?

**DOI:** 10.3390/molecules25133060

**Published:** 2020-07-04

**Authors:** Anna Kicinska, Wieslawa Jarmuszkiewicz

**Affiliations:** Department of Bioenergetics, Faculty of Biology, Adam Mickiewicz University, Poznan, Uniwersytetu Poznanskiego 6, 61-614 Poznan, Poland; wiesiaj@amu.edu.pl

**Keywords:** flavonoids, mitochondria, cytoprotection, mitochondrial ion channels

## Abstract

A large number of diverse mechanisms that lead to cytoprotection have been described to date. Perhaps, not surprisingly, the role of mitochondria in these phenomena is notable. In addition to being metabolic centers, due to their role in cell catabolism, ATP synthesis, and biosynthesis these organelles are triggers and/or end-effectors of a large number of signaling pathways. Their role in the regulation of the intrinsic apoptotic pathway, calcium homeostasis, and reactive oxygen species signaling is well documented. In this review, we aim to characterize the prospects of influencing cytoprotective mitochondrial signaling routes by natural substances of plant origin, namely, flavonoids (e.g., flavanones, flavones, flavonols, flavan-3-ols, anthocyanidins, and isoflavones). Flavonoids are a family of widely distributed plant secondary metabolites known for their beneficial effects on human health and are widely applied in traditional medicine. Their pharmacological characteristics include antioxidative, anticarcinogenic, anti-inflammatory, antibacterial, and antidiabetic properties. Here, we focus on presenting mitochondria-mediated cytoprotection against various insults. Thus, the role of flavonoids as antioxidants and modulators of antioxidant cellular response, apoptosis, mitochondrial biogenesis, autophagy, and fission and fusion is reported. Finally, an emerging field of flavonoid-mediated changes in the activity of mitochondrial ion channels and their role in cytoprotection is outlined.

## 1. Introduction

Different forms of cell death (i.e., apoptosis, necroptosis, ferroptosis, and necrosis) have been implicated in the pathogenesis of many human diseases. Myocardial infarction, myocardial infarction with reperfusion, stroke and Alzheimer’s disease are perhaps the most important examples in terms of mortality, morbidity, and cost. Ischemic heart disease alone accounts for 20% of deaths in the European Union [1]. Importantly, protecting cardiomyocytes from death resulting from acute myocardial infarction is vital for improving survival. It has been demonstrated that infarct size remains a major determinant of subsequent heart failure and mortality [2,3]. Additionally, in the case of hemorrhagic and ischemic stroke, extensive neuronal death is known to follow the event [4]. The death of neuronal cells is also implicated in the neurogenerative diseases Alzheimer’s and Parkinson’s disease, both of which have shown increasing prevalence in aging populations [5,6]. Cell death restriction is also vital for the moderation of severe complications that accompany many other conditions, ranging from oxidative damage, obesity, inflammation, etc. to toxicity of chemotherapeutic agents [7,8,9,10].

More than 9000 flavonoids are known to be present in plants and several hundred of them are ubiquitous in the human diet. The reported total consumption of flavonoids by adults ranges from 209 to 1017 mg/d in European, U.S., and Australian cohort studies [11]. Moreover, it has been shown that the level of dietary flavonoid consumption correlates with the reduced risk of many noncommunicable diseases in epidemiological, preclinical, and clinical studies [12]. The effect has been very recently described by Bondonno et al. in a Danish Diet, Cancer, and Health cohort study involving 56,048 participants and 23 years of follow-up [13]. Namely, the moderate intake of flavonoids has been shown to be inversely associated with cancer-related, cardiovascular disease-related, and all-cause mortality, especially for individuals who smoke or consume large amounts of alcohol. Extensive studies have shown that flavonoids have antioxidant [14,15], metal chelation [16,17], signal transduction, gene expression, and enzyme function modulating properties [18]. The potential therapeutic application of flavonoids has been studied in the context of the prevention and treatment of cardiovascular disease, diabetes, cancer, and cognitive diseases. Indeed, it has been demonstrated that flavonoids, i.e., suppress the expression of pro-inflammatory mediators (NF-κβ cascade), have vasodilator activity, improve vascular endothelial function, protect cells against insulin resistance, regulate proliferation, and suppress neuroinflammation by reducing cytokine release [19,20,21,22,23,24,25,26]. As shown in many studies, the consumption of flavonoid-rich foods significantly decreases the possibility of cardiovascular disease development [11,27,28,29]. A recent meta-analysis demonstrated that dietary intake of anthocyanins reduced the risk of coronary heart disease and cardiovascular disease mortality [30]. Other studies have shown a significant reduction in systolic and diastolic blood pressure by, i.e., cocoa flavanols [31,32]. The improvement of endothelial function and thus, the prevention of vasoconstriction have also been demonstrated [12]. Additionally, the incidence of stroke was reduced with increased dietary flavonol intake [33]. Data on neurodegenerative disorders are also encouraging [34,35]. The results by Shishtar et al. suggest that higher long-term consumption of flavonoids in the diet lowers the risk of Alzheimer’s disease development in adults [34]. Parkinson’s disease progression in men is also less likely with higher dietary flavonoid consumption [36]. Recently, anthocyanins and flavan-3-ols have been shown to reduce the risk of type II diabetes mellitus [37]. In addition, interest in the interaction of flavonoids with gut microbiota is increasing [38]. The influence on gut microbiome constituents and their function has been described. This effect leads to the modulation of endotoxin production, primary to secondary bile acid conversion, gut immune homeostasis, and nutrient absorption and metabolism [39].

The role of mitochondria in the sustenance of cellular functions is clear, as these organelles are at the center of cellular metabolism and signaling pathways. In this review, we intend to describe in detail the interaction of flavonoids with mitochondrial pathways. Our aim is to concentrate on the modulation of mitochondrial function by flavonoids, which leads to cytoprotection and thus the possible application of these chemicals as pharmacological agents.

## 2. Flavonoids

**Figure 1:**
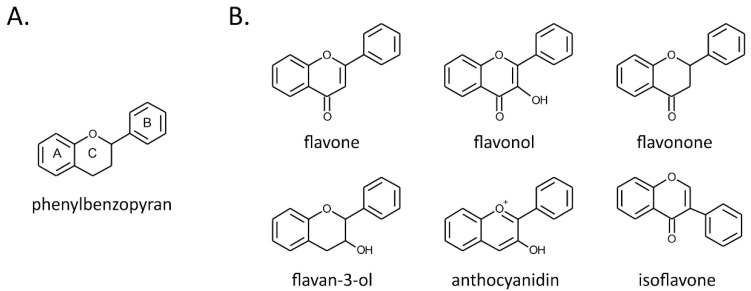
Flavonoid structures. Chemical structures of phenylbenzopyran (**A**) and flavonoid classes (**B**).

Flavonoids represent a range of polyphenolic plant secondary metabolites. They share a common phenylbenzopyran structure with two benzene rings linked by a heterocyclic pyran ring (Figure 1A) [40]. Classes of flavonoids are defined by the level of oxidation and saturation of the C ring. The major classes are flavanones, flavones, flavonols, flavan-3-ols, anthocyanidins, and isoflavones. Within the classes, the compounds differ in the pattern of substitutions of A and B rings (Figure 1B). Flavonoids are widely distributed among plants and are crucial for various aspects of plant interactions with the environment. They attract pollinating insects [41], protect plants against UV, act as antimicrobial agents, and deter herbivores [42,43,44]. Due to the numerous reports suggesting the beneficial effects of flavonoids on human health, there has been continuously growing interest in their nutritional role. The most common dietary sources of flavonoids for humans are berries, grapes, teas, cocoa-based products, apples, onions, parsley, citrus fruits, and soybeans (Table 1) [45,46,47,48,49]. After being consumed, flavonoids undergo extensive transformations, leading to their absorption, distribution, metabolism, and finally elimination [50]. In the epithelium and in the lumen of the small intestine, flavonoids in glycosidic forms are hydrolyzed. The aglycone forms are then transported to the liver. In the liver, they are oxidized or demethylated by phase I drug-metabolizing enzymes. Further rapid glucuronidation, sulfation, or methylation by phase II drug-conjugating enzymes takes place in the liver or intestine. However, a large proportion (up to 90%) of dietary flavonoids are not absorbed in the small intestine. These compounds have been found to be metabolized in the large intestine by microbiota into absorbable aglycone forms or other biologically active small metabolites, e.g., ring-fission products [51].

## 3. Mitochondrial Pathways

**Figure 2:**
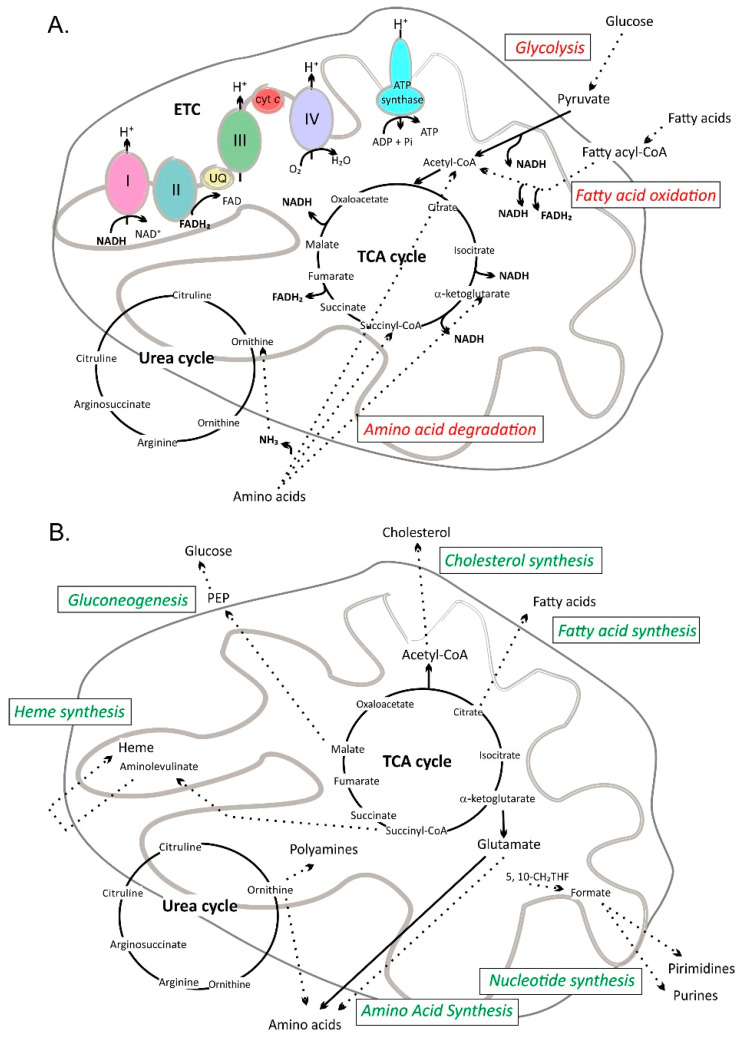
Mitochondrial catabolic and anabolic pathways. Pyruvate, fatty acid, and amino acid oxidation is accompanied by a reduction of NAD^+^ and FAD. The electrons from NADH and FADH_2_ are transferred to the electron transport chain (ETC) and the proton electrochemical gradient is built that drives ATP synthesis. (**A**). Mitochondria as a source of building blocks for biosynthesis. The tricarboxylic acid cycle (TCA cycle) and urea cycle supply metabolites for synthesis of glucose, amino acids, heme, cholesterol, and fatty acids. Nucleotide synthesis also partially takes place in mitochondria (**B**). Dotted line—multiple reaction steps; PEP—phosphoenolpyruvate; UQ—ubiquinone; 5,10-CH_2_THF—5,10-methylenetetrahydrofolate; I, II, III, IV—respiratory chain complexes; cyt *c*—cytochrome *c.*

**Figure 3:**
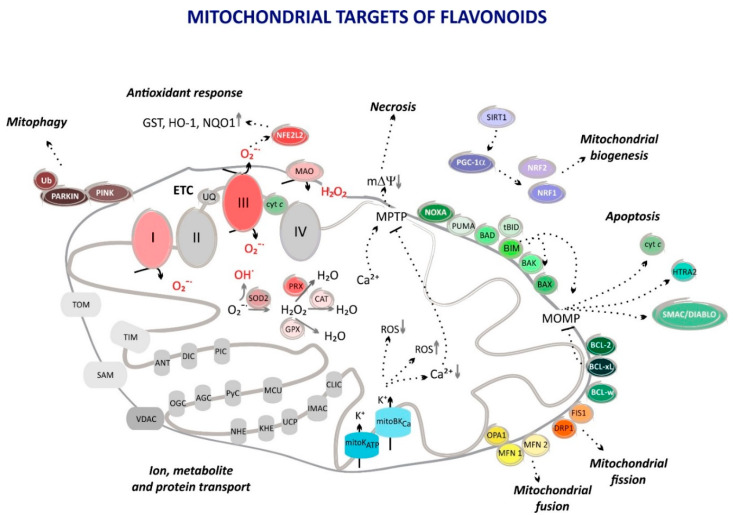
Mitochondrial targets of flavonoids. Mitochondrial regulation of apoptosis, necrosis, antioxidant response, and mitochondrial dynamics. The influence of flavonoids on the pathways marked in color is described in this review. The effect of flavonoids on antioxidant response is mediated by modulating antioxidant enzymes hemeoxygenase 1 (HO-1), NADPH:quinone oxidoreductase 1 (NQO1), superoxide dismutase (SOD2), peroxiredoxin (PRX), glutathione peroxidase (GPX), catalase (CAT) and ROS generation by complex I (I) and complex III (III) of the electron transport chain (ETC), and monoamine oxidase (MAO). Flavonoids regulate apoptosis by changing the levels of pro-(BAX, BIM, BAD, PUMA, NOXA, and BID) and anti-apoptotic proteins (BCL-2, BCL-XL, BCL-w). Mitochondrial dynamics is affected by tuning mitochondrial biogenesis (sirtuin—SIRT1; PGC-1α—proliferator-activated receptor gamma coactivator; NRF1, NRF2—nuclear respiratory factor 1, 2), fission (mitochondrial fission protein 1—FIS1; dynamin-related protein 1—DRP1), fusion (mitofusin 1—MFN1; mitofusin 2—MFN2; optic atrophy protein 1—OPA-1), and mitophagy (PINK and PARKIN). The flavonoid interaction with potassium channels of the inner mitochondrial membrane (mitochondrial ATP-sensitive K^+^ channel—mitoK_ATP_; mitochondrial large conductance K^+^ channel K_Ca_1.1—mitoBK_Ca_) results in an increased K^+^ flux into mitochondria. In addition, the proteins involved in transport of ions and metabolites through mitochondrial membranes are presented. AGC—aspartate–glutamate carrier; ANT—adenine nucleotide translocase; CLIC—chloride intracellular channel; cyt *c*—cytochrome *c*; DIC—dicarboxylate carrier; HTRA2—high-temperature requirement protein A2; IMAC—inner membrane anion channel; KHE—K^+^/H^+^ exchanger; mΔΨ—mitochondrial inner membrane potential; MCU—mitochondrial calcium uniporter; MOMP—mitochondrial outer membrane permeabilization; MPTP—mitochondrial permeability transition pore; NF2L2—nuclear factor erythroid 2-related factor 2; NHE—Na^+^/H^+^ exchanger; OGC—2-oxoglutarate carrier; PIC—phosphate carrier; PyC—pyruvate carrier; SAM—sorting and assembly machinery; TIM—translocase complex of the inner membrane; TOM—translocase complex of the outer membrane complex; Ub—ubiquitin; UCP—uncoupling protein; VDAC—voltage dependent anion channel.

Mitochondria are complex and multifunctional organelles engaged in almost all cellular processes [52,53]. They are well known for being a cellular “powerhouse” due to their role in the catabolism of carbon-rich fuel molecules (glucose, lipids, and glutamine) and in ATP synthesis via oxidative phosphorylation (OXPHOS). ATP synthase, located in the inner mitochondrial membrane, uses a proton electrochemical gradient produced by electron transport chain (ETC) complexes to drive ATP synthesis. The reduction of necessary electron carriers (NAD^+^ and FAD) takes place in the mitochondrial matrix via the tricarboxylic acid (TCA) cycle (Figure 2A). Other equally fundamental metabolic processes (amino acid catabolism, fatty acid oxidation, the urea cycle, elements of the biosynthetic pathways leading to fatty acids, cholesterol, nucleotides, amino acids, glucose, and heme) also occur in mitochondria (Figure 2B). Moreover, these organelles are an essential part of cellular signaling pathways, both as end effectors responsive to changes in energy demand and as initiators and transducers [54,55] (Figure 3). Mitochondria play a central role in calcium homeostasis [56], influencing, among many other processes, the release of neurotransmitters and hormones [57,58], tissue regeneration [59], and interferon-β signaling [60]. As a main source of cellular reactive oxygen species (ROS), mitochondria are involved in ROS signaling [61,62,63]. Alterations in the level of mitochondria-produced ROS have been found to modify, e.g., immune system function [64], angiotensin II signaling [65], insulin secretion [66], and various stress responses [67,68]. Important elements of cellular ROS scavenging systems are also located in mitochondria. These include superoxide dismutase (SOD2), glutathione peroxidases (GPX 1 and GPX4), peroxiredoxins (PRX3, PRX5), thioredoxin 2, and thioredoxin reductase 2 (TRR2) [69]. Mitochondria are also part of multiple pathways that lead to cell death. The intrinsic apoptotic pathway is initiated in response to a large number of stimuli in all multicellular organisms by mitochondria-derived factors [70]. Cytochrome *c*, the second mitochondria-derived activator of caspase (SMAC/DIABLO) and OMI/high-temperature requirement protein A2 (HTRA2) promote procaspase activation. The release of cytochrome *c*, SMAC/DIABLO and HTRA2 from mitochondria requires mitochondrial outer membrane permeabilization (MOMP). MOMP is tightly regulated by pro- and anti-apoptotic BCL-2 family proteins (BAX, BAK, BIM, BID, PUMA, BAD, NOXA, etc. and BCL-2, BCL-xL, BCL-w, respectively) [71,72]. Mitochondria also contribute to the extrinsic apoptotic pathway by amplifying the death signal, e.g., by BID cleavage by Caspase-8 leading to MOMP and the release of mitochondrial factors [72,73]. Regulated necrosis [74], ferroptosis [75] and parthanatos [76] are additional cell death types in which mitochondria play an important role. Moreover, a crucial event in necrosis, i.e., the Ca^2+^-induced opening of the mitochondrial permeability transition pore (MPTP), is also mediated by mitochondria [77]. This event leads to the rapid dissipation of mitochondrial inner membrane potential (mΔΨ) and ATP depletion.

Efficient and regulated transport across the outer and inner mitochondrial membranes (OMM and IMM, respectively) is required for mitochondrial function. OMM is equipped with a number of channel-forming proteins that show quite broad substrate specificity. The transport of proteins, small hydrophilic ions and metabolites is mediated by the translocase of the outer membrane (TOM) complex, the sorting and assembly machinery (SAM) complex, Mdm10, Mim and the voltage-dependent anion channel (VDAC) [78]. The IMM, which maintains the bioenergetic functions of mitochondria must be impermeable to protons. However, it contains proteins responsible for the transport of other cations (K^+^, Na^+^, Mg^+^, and Ca^2+^) and anions (nucleotide phosphates, di- and tricarboxylates, Cl^−^, and PO_4_^−3^). The transport of metabolites and ions across the IMM is tightly regulated and mediated by numerous specific metabolite carriers, translocases, and ion channels [79,80]. Over the last 30 years, the existence of ion channels in the IMM has attracted growing appreciation and interest [81,82,83,84,85]. A large variety of cation- and anion-selective channels have been described. For example, potassium channels similar to all types of K^+^ channels previously discovered in the plasma membrane (inwardly rectifying, two P-domain, voltage-gated, and calcium- and sodium-regulated potassium channels) have been found in the IMM [81,86]. The inner membrane anion channel (IMAC) and chloride intracellular channel proteins 4 and 5 (CLIC4 and CLIC5) represent the anion channel family of the IMM [82,86]. Since the discovery in 1997 that the activation of the mitochondrial ATP-sensitive K^+^ channel (mitoK_ATP_) protects hearts against ischemia/reperfusion (I/R) injury [87,88], an important role of IMM K^+^ permeability in cytoprotection has been described in numerous cellular models and various insults [89]. It all started with the discovery that the activation of the mitoK_ATP_ channel mimics ischemic preconditioning (IPC)—a well-known phenomenon in which brief periods of ischemia protect cells against the subsequent injury resulting from sustained ischemia [90,91]. Since then, it has been shown that the involvement of mitoK_ATP_ channel activation provides protection in brain, heart and muscle cells [92,93]. The activation of the mitochondrial large conductance K^+^ channel K_Ca_1.1 (mitoBK_Ca_) has also been described as cytoprotective, for instance, in the heart and brain [85,94,95,96,97,98]. The mechanisms involved in the cytoprotection triggered by K^+^ transport activation in the IMM are not yet fully understood. However, it has been suggested that these mechanisms may involve the induction of mitochondrial ROS production and triggering prosurvival pathways or, on the contrary, may involve lowering ROS levels during reperfusion [99,100,101,102]. The decrease in the massive Ca^2+^ influx into the mitochondrial matrix at reperfusion due to mild uncoupling after K^+^ channel activation may also diminish the possibility of MPTP opening and cell death [103]. Mitochondrial volume regulation, and thus the regulation of ATP synthesis efficiency, have also been described as part of the mechanisms [104].

## 4. Flavonoids in Mitochondrial Pathways

### 4.1. Flavonoids as Mitochondrial ROS Scavengers

Increased ROS levels cause the oxidation of proteins, nucleic acids, and lipids leading to detrimental processes, such as cellular aging [105], mutagenesis [106], and carcinogenesis [107]. The beneficial effects of flavonoids on health have long been attributed to their antioxidant properties [41], which lead to a reduction in ROS, regardless of their source (endogenous: mitochondria, peroxisomes, xanthine oxidase, Fenton reaction, NADPH oxidase, lipoxygenases, cytochrome P450 or exogenous: visible, UV and ionizing radiation, chemotherapeutics, etc.) [108,109]. The ability to scavenge ROS and reactive nitrogen species (RNS) is determined by the hydroxyl configuration of the flavonoid B-ring, as it donates hydrogen and an electron to superoxide (O_2_^−**•**^) [110], hydroxyl (**^•^**OH), peroxyl (ROO**^•^**) and peroxynitrite (ONOO^−^) [111]. Relatively stable flavonoid radicals are formed in this process [111]. The direct antioxidant effect of numerous flavonoids has been demonstrated in many studies using in vitro and ex vivo models in cell cultures, tissue homogenates, etc., as well as in vitro in animal models [112]. As mitochondria are the main source of intracellular ROS, flavonoid antioxidant effects are briefly described in this chapter. For example, flavonoids from red wine significantly reduce the oxidation of low-density lipoprotein in humans [113], baicalein binds iron ions and strongly inhibits the Fenton reaction by ROS scavenging in combination with iron chelation [114], flavonoids have also been found to protect rat hippocampal cells against oxidative stress by ROS scavenging [115], whereas silibinin A protects neuronal and liver cells from nitrosative stress by influencing mitochondria, namely, increasing mΔΨ and ATP levels [116]. A recent study showed the direct scavenging of O_2_^−**•**^ generated in mitochondrial complex III of the ETC by quercetin in isolated rat heart mitochondria [117]. Numerous studies show that flavonols (catechins and quercetin) protect cells against oxidative injury by activating the transcription of antioxidant enzymes in nuclear factor erythroid 2-related factor 2 and an antioxidant response element (ARE)-dependent manner [118,119,120,121,122]. Enzymes involved in this regulation of cellular redox status and protection against oxidative damage include glutathione-*S*-transferase (GST), hemeoxygenase 1 (HO-1) and NADPH:quinone oxidoreductase 1 (NQO1). The antioxidant properties of flavonoids have also been shown to improve cell survival in cerebral I/R injury [123], colistin-induced nephrotoxicity [124], chronic inflammatory diseases [125] and Parkinson’s disease [126]. In addition, flavonoids have also been shown to modulate cellular endogenous levels of antioxidants by influencing the activity of enzymes responsible for glutathione synthesis (e.g., glutathione reductase) and antioxidant enzymes (glutathione peroxidase, superoxide dismutase, and catalase) [127,128]. Manuka honey, containing a mixture of polyphenols, including flavonoids, has been shown to protect skin fibroblasts against oxidative stress and improve wound healing by ameliorating mitochondrial function and induction of superoxide dismutase and catalase [122]. Although there are reports questioning the direct role of flavonoids in ROS scavenging (since plasma concentrations of flavonoids after indigestion are approximately two orders of magnitude lower than an effective antioxidant dose in vitro-IC_50_ 10–100 µM) [129], they may exert their protective actions in exposed organs such as gastrointestinal or skin mucosa or after specific accumulation in mitochondria [128]. Indeed, mitochondria-targeted ROS scavengers have been demonstrated to be protective in certain types of cancer and cardiac disease [69,130].

### 4.2. Flavonoids Attenuate Mitochondrial ROS Formation

Mitochondrial complex I (NADH:ubiquinone oxidoreductase) and complex III (ubiquinol: cytochrome *c* oxidoreductase) of the ETC are the most prominent sources of ROS, which are generated as a byproduct of electron transfer [63,69]. Flavonoids have been found to suppress ROS production by directly inhibiting enzymes and chelating the trace elements involved in ROS generation [131]. Early studies showed that flavonoids, namely, luteolin, myricetin, fisetin, robinetin, rhamnetin, and baicalein, inhibit complex I [132,133]. Other reports describe complex III inhibition by hispidulin and eupafolin [134,135]. In isolated mitochondria, reduced complex I activity has been linked to reduced ROS generation. Inhibition of complex I activity by quercetin, kaempferol, and epicatechin has been found to significantly lower H_2_O_2_ production in isolated rat heart mitochondria [136]. Competition between flavonoids and ubiquinone for close binding sites has been suggested. Catechin also inhibits complex I in isolated rat heart mitochondria and decreases H_2_O_2_ generation [137]. Nobiletin decreases mitochondrial oxygen consumption and H_2_O_2_ production in the presence of glutamate and malate, but a slight increase has been observed with succinate [138]. However, other reports have shown that the inhibition of complex I by an apple peel polyphenol extract leads to increased mitochondrial superoxide production [139]. In addition, the reduction in mitochondrial ROS generation by flavonoids was associated with slight uncoupling properties of some of these substances, i.e., galangin [140]. The reduction in mΔΨ resulting from increased H^+^ flow into the mitochondrial matrix diminishes ROS formation. It has also been shown that an alternative mitochondrial source of ROS, namely, mitochondrial membrane-bound monoamine oxidase (MAO), is inhibited by numerous flavonoids [128,141]. MAO is implicated in neurodegeneration because its overexpression increases **^•^**OH generation, causing oxidative stress and neuronal death.

### 4.3. Antiapoptotic Substances

Although numerous reports have described the proapoptotic effects of flavonoids and thus their possible use in cancer prevention [142,143], several flavonoids have also been shown to have potent antiapoptotic activity and protect cells against damage caused by various stimuli, both in vitro and in vivo in animal disease models. The involved mechanisms include the inhibition of the intrinsic apoptotic pathway by the preservation of mitochondrial function (i.e., maintaining mΔΨ), regulation of redox potential and MOMP by downregulation of BAX and BAK or upregulation of BCL-2 and BCL-xL [144]. In the case of I/R-induced cell death, an increase in the protein level ratio of antiapoptotic BCL-2/proapoptotic BAX has been induced in numerous experimental models by different flavonoids, i.e., tilinin, luteolin, *Bauhinia championii* flavone (in myocardium), quercetin (in bladder and PC12 cells), baicalein (in the lungs and brain), and apigenin and naringenin (in the brain) [145,146,147,148,149,150,151,152]. A similar mechanism has been described for quercetin in oxidative stress-treated PC12 cells, as well as for chrisin in Parkinson’s disease models [153,154]. Moreover, in PC-12 cells, hesperidin has been found to protect against amyloid-β(Aβ)-induced apoptosis by reversing Aβ-induced mitochondrial dysfunction and leading to a decrease in MPTP opening and an increase in cell survival [155]. It has also been observed that cytotoxic substance-induced apoptosis is reduced by flavonoids. Namely, arsenic-induced apoptosis is alleviated by (−)-epigallocatechin-3-gallate [156]. The mechanisms involved include mΔΨ preservation and apoptosis inhibition. Similarly, a number of reports show the antiapoptotic regulation of BCL-2 family protein expression, i.e., diosmetin in endotoxin-induced hepatotoxicity, anthocyanin in gentamycin-induced hepatotoxicity, apigenin and kaempferol in doxorubicin-induced cardiotoxicity and nobiletin in cisplatin-induced kidney injury [157,158,159,160,161].

### 4.4. Influence on Mitochondrial Biogenesis

The tightly regulated process of the generation of new mitochondria (mitochondrial biogenesis) together with the removal of damaged mitochondria (mitophagy) preserves mitochondrial homeostasis [162]. The stimulation of mitochondrial biogenesis is important as an adaptive mechanism in the cellular response to different stressors. Mitochondrial biogenesis also seems to be a promising therapeutic target as it provides a protective mechanism in a broad spectrum of acute and chronic diseases manifested by mitochondrial dysfunction [163]. Mitochondrial biogenesis is coordinated by specific nuclear transcription factors. The major elements in the network are proliferator-activated receptor gamma coactivators (PGC-1α and PGC-1β) and nuclear respiratory factors (NRF1 and NRF2). The pathway is initiated by the activation of PGC-1α by phosphorylation or deacetylation, which leads to the stimulation of a series of transcription factors, including NRF1, NRF2 and estrogen-related-α (ERR-α) [164]. NRF1 regulates the transcription of genes encoding many mitochondrial proteins, as well as transcription factors responsible for mtDNA transcription, including transcription factor A mitochondrial (TFAM) and transcription factor B1 mitochondrial (TFB1M), among others. Upstream of PGC-1α, there are multiple regulators, including sirtuin (SIRT1), 5′ adenosine monophosphate-activated protein kinase (AMPK), cAMP-response element binding protein (CREB) and forkhead transcription factor (FOXO1) [165]. Flavonoids belonging to almost all classes have been found to stimulate mitochondrial biogenesis in various experimental models. Most of these studies, carried out both in vitro and in vivo, show that upregulation of PGC-1α is a central phenomenon in these processes. Rasbach and Schnellmann demonstrated that isoflavones increase mitochondrial biogenesis by stimulating PGC-1α expression and SIRT1 expression and/or activity [166]. Quercetin has also been shown to induce mitochondrial biogenesis in the muscle and brain of mice in vivo. Increases in SIRT1 and PGC1α expression and mtDNA copy number were observed [167]. Nieman et al. showed that 2 weeks of quercetin supplementation resulted in a significant increase in skeletal muscle mRNA levels of SIRT1, PGC-1α, cytochrome *c* oxidase, citrate synthase, and the relative copy number of muscle mtDNA in untrained young adult males [168]. The induction of the expression of PGC-1α, NRF-1, TFAM, mtDNA, and mitochondrial proteins in HepG2 cells by quercetin has been described [169]. These effects seem to be heme oxygenase/carbon monoxide dependent. Yoshimo et al. have shown using a C2C12 murine muscle cell line in an in vitro model that flavonoids (with the most potent being isoflavone daidzein) directly activate the TFAM promoter [170]. The effect was again PGC-1α, NRF, and SIRT1 dependent. More recent studies have shown PGC-1α-, SIRT1-, and/or AMPK-dependent effects of myricetin and tangeretin on mitochondrial biogenesis in murine skeletal muscles and of isoharmnetin in adipocytes [171,172,173]. In addition, flavonoid-induced protection has been demonstrated in various models of human diseases. For example, the induction of mitochondrial biogenesis by baicalein reverses mitochondrial dysfunction in a Parkinson’s disease model, puerarin improves mitochondrial performance in diabetic rats, quercetin is beneficial in osteoarthritis and traumatic brain injury models, and dihydromycetin ameliorates cardiac I/R injury [174,175,176,177,178].

### 4.5. Mitochondrial Autophagy Regulators

Mitochondrial autophagy or mitophagy is a process of mitochondrial degeneration in which mitochondrial remnants are transported to peroxisomes or lysosomes for degradation. In most mammalian cell types, mitochondrial impairment leads to the stabilization of phosphatase and tensin homolog-induced kinase 1 (PINK1) on the outer mitochondrial membrane [179]. This leads to the phosphorylation of ubiquitin and the recruitment of the E3 ubiquitin ligase—Parkin. Mitochondrial proteins are then polyubiquitinated, and an autophagosome is formed, which next fuses with the lysosome for degradation. In 2012, Filomeni et al. demonstrated that kaempferol treatment restores the impaired mitophagy induced by acute rotenone toxicity and that the enhancement of mitochondrial turnover is crucial for cell survival [180]. Quercetin has also been found to alleviate the mitochondrial damage induced in murine liver by chronic ethanol treatment by inducing Parkin-dependent mitophagy [181]. More recent studies have shown that quercetin enhances PINK1/Parkin-dependent mitophagy in a nonalcoholic fatty liver disease model [182]. Interestingly, the activation of mitophagy is mediated by the stimulation of frataxin expression. Frataxin is a mitochondrial protein involved in assembly of iron-sulfur cluster-containing proteins. Purerarin, in addition to showing other beneficial effects on palmitate-induced mitochondrial dysfunction, has been shown to attenuate impaired mitophagy via the PINK1/Parkin pathway [183]. In contrast, naringin has been shown to have protective effects during cerebral I/R injury by inhibiting the translocation of Parkin into mitochondria and thus ONOO^-^-mediated excessive mitophagy [184].

### 4.6. Mitochondrial Fission and Fusion Control

Rather than being static and fixed structures, mitochondria are dynamic organelles that undergo constant morphological changes. Their structure constantly adapts via fusion and fission events to meet cellular needs, including metabolic demands and nutrient availability (for a recent review see [185]). Fusion, which means joining mitochondria together to form a tubular network, possibly allows matrix components to be distributed, improves oxidative phosphorylation efficiency, and prevents autophagy. On the other hand, fission, which is the division of a mitochondrion, is enhanced under stress conditions (associated with mitochondrial dysfunction) and in the G2/M phase of the cell cycle. The proteins important for the correct progression of these processes include dynamin-related protein 1 (DRP1), dynamin 2 (DNM2), and mitochondrial fission protein 1 (FIS1) in fission [186] and mitofusins 1 and 2 (MFN1, MFN2) and optic atrophy protein 1 (OPA1) in fusion [187]. Recent reports show that various flavonoids are able to regulate mitochondrial dynamics. Quercetin has been described to protect against acute hypobaric hypoxia-induced mitochondrial dysfunction in rats by inducing mitochondrial biogenesis via the SIRT1, PGC-1α, TFAM, and NRF1 pathways, and by inhibiting mitochondrial fission [188]. The expression of DRP1 and FIS1 was enhanced by hypobaric hypoxia and significantly reduced after quercetin treatment. At the same time, the expression levels of MFN1 and MFN2 were restored by quercetin, indicating the protection of mitochondria against excessive stress-induced fission and reduced fusion. Similar effects of quercetin have been described in a model of adenine-induced aortic calcification. Decreased expression and phosphorylation of DRP1 suppressed mitochondrial fission [189]. The mechanism of the quercetin-induced alleviation of mitochondrial fragmentation has been recently unraveled in murine endothelium [190]. Chen et al. showed that quercetin specifically inhibits DRP1 phosphorylation at Ser 616, possibly by inhibiting PKCδ [190]. Parrado-Fernandez et al. also showed that anthocyanins prevent mitochondrial fragmentation in rotenone- or familial Alzheimer’s disease genetic mutation-induced models of Alzheimer’s disease in SH-SY5Y cells [191]. Anthocyanins block DRP1 overexpression and restore the expression levels of MFN2 in cells carrying the mutation. Grape seed proanthocyanidins also prevent mitochondrial damage in irradiated human lung fibroblasts by regulating DRP1 and MFN1 and 2 expression levels [192]. Baicalin has also been reported to suppress mitochondrial fission and enhance fusion by lowering the expression of DRP1 and stimulating the expression of MFN2 [193]. One of the important effects of kaempferol in the neuronal ischemic stroke model was the suppression of DRP1 activation [194]. Dihydromyricetin attenuated dexamethasone-induced muscle atrophy by sustaining mitochondrial function, among other effects, inducing fusion by stimulating MFN2 expression [195]. Additionally, puerarin and quercetogetin have been described to protect cells against palmitate- and cigarette smoke extract-induced mitochondrial dysfunction by regulating mitochondrial dynamics [183,196].

### 4.7. Mitochondrial Ion Channel Openers

In 2006, Gao et al. showed that puerarin diminishes IR-induced injury in isolated rat hearts and that the effect is mitoK_ATP_ channel dependent [197]. Inhibition of channel activity by 5-hydroxydecanoate (5-HD) abolishes viability and hemodynamic function improvement. Genistein has also been demonstrated to be cardioprotective in a rabbit model of IR injury, where its intravenous injection after coronary artery occlusion (prior to reperfusion) reduces infarct size [198]. The effect was again reversed by 5-HD. Interestingly, Akt phosphorylation is involved in the process, and the simultaneous inhibition of glycogen synthase kinase 3β provides protection in the case of extended ischemia [198]. Proanthocyanidins protect neonatal rat myocardial cells against anoxia/reoxygenation by increasing survival, diminishing ROS production, activating Caspase-3, and promoting Akt phosphorylation [199]. Again, all these effects are dependent on the activation of the mitoK_ATP_ channel and reversed by 5-HD. The same is true for naringenin applied during an I/R injury protocol with isolated rat hearts. The flavonoid significantly decreases the infarct area and reduces cell death. All effects are sensitive to glibenclamide (another K_ATP_ channel inhibitor) and 5-HD [200]. The works of Testai et al. starting in 2013 implicate the role of mitoBK_Ca_ channel activation in the naringenin effect [201]. In a model of acute infarct in rats, naringenin reduces IR-induced heart injury. The effect is reversed by paxilline, a mitoBK_Ca_ channel inhibitor. Furthermore, in a perfused heart model, naringenin improves postischemic functional parameters and decreases myocardial injury. A direct influence of naringenin on mitochondria revealed by mΔΨ depolarization and reduction of Ca^2+^ accumulation in mitochondrial matrix has been observed in this model [201]. Moreover, naringenin cardioprotection has been characterized in vivo and ex vivo in aging rats [202]. The naringenin-induced effects observed in this study were again antagonized by paxilline. In 2019, Kampa et al. finally showed the direct interaction of the flavonoid naringenin with mitochondrial potassium channels [203]. It has been demonstrated using isolated mitoplasts from primary human dermal fibroblasts and patch-clamp that naringenin at micromolar concentrations directly increases mitoBK_Ca_ and mitoK_ATP_ channel activity. Moreover, naringenin-induced activation of mitochondrial K^+^ channels leads to mild mitochondrial uncoupling and results, as expected, in an increase in the mitochondrial respiration rate in these cells. In addition, our latest study indicates that in mitochondria isolated from human endothelial cells, naringenin stimulates inhibitor-sensitive mitoBK_Ca_ channel-mediated K^+^ flux, decreasing the mΔΨ and thus accelerating the mitochondrial oxygen consumption rate [204]. We have also demonstrated using patch clamp that naringenin directly activates the mitoBK_Ca_ channel from endothelial mitochondria. Furthermore, naringenin prevents cell damage in this model [204]. Thus, mitochondrial ion channels are becoming promising new targets for flavonoids in cells.

## 5. Conclusions and Perspectives

A large amount of scientific evidence showing the cytoprotective and possibly therapeutic application of numerous flavonoids in human diseases is available. In the case of civilization diseases that affect large populations, diet-based medicine seems to be extremely beneficial. The use of bioactive substances of natural origin could contribute to cost-effective disease prevention and could improve and reduce observed side-effects of conventional therapies. In this review, we have presented flavonoids as modifiers of mitochondrial function, substances that prevent mitochondrial damage resulting from many insults and subsequent cell dysfunction. In addition to the direct effects of mitochondrial ROS scavenging, we have described the modulation of mitochondrial ROS generation, mitochondrial antioxidant system activity, mitochondrial apoptotic pathway, biogenesis, mitophagy, fission and fusion, and mitochondrial potassium channel activity. The most representative examples of reported flavonoids and their cytoprotective properties in various models are summarized in Table 2.

However, certain issues remain to be addressed. In this review, we focused on mitochondria-mediated effects, but flavonoids seem to affect almost all cell signaling routes. Experimental and methodological issues make it more difficult to identify pathways on which flavonoids have no effect than to identify those on which flavonoids act. It is very important to determine the exact sites of flavonoid interaction in cells and thus to distinguish between direct and indirect effects and to understand the underlying complexity. The effects defined for a given disease model could lead to unpredictable results in others. The inhibition of cellular death notably benefits in cardiac I/R-induced injury but is detrimental in cancer. It is believed that there is no flavonoid toxicity, but some reports indicate that further studies are inevitable because some flavonoids has been observed to have pro-oxidative, estrogenic, or carcinogenic potential [205]. In addition, vast heterogeneity has been observed in the individual response to increased uptake. The variability in absorption and metabolism must be accounted for.

## Figures and Tables

**Table 1:** Flavonoid classes and their representative dietary sources [47,48,49].

Flavonoid Class	Selected Compounds	Examples of Dietary Sources
Flavanones	Hesperetin	Oranges, tangelo, lemons, limes
	Naringenin	Grapefruit, pomelo, kumquats, oregano
	Eriodictyol	Oregano, peppermint, oranges, lemons
Flavones	Apigenin	Parsley, celery, kumquats
	Baicalein	Welsh onion, Chinese skullcap
	Luteolin	Peppers, radicchio, oregano, celery seed
	Tangeretin	Tangerines, sweet oranges
Flavonols	Fisetin	Strawberry, apple, persimmon, grape, onion
	Kaempferol	Capers, saffron, arugula, chard, chives
	Myricetin	Cranberries, goji berry
	Quercetin	Capers, elderberry, chokeberry
Flavan-3-ols	Catechin	Cocoa, green tea, blueberries, blackberries
	Epicatechin	Cocoa, green tea, grapes, red wine
	Epigallocatechin	Green tea, apples, plums, nuts
Anthocyanidins	Cyanidin	Chokeberries, elderberries, blackberries, red cabbage
	Delphinidin	Blackcurrants, blueberries, grapes
	Pelargonidin	Strawberries, radishes
Isoflavones	Genistein	Soy, red clover, alfalfa
	Daidzein	Soy, nuts
	Glycitein	Soy

**Table 2:** Representative examples of flavonoids and their cytoprotective effects on mitochondrial pathways in various models. I/R—ischemia/reperfusion; ETC—electron transport chain; TNF-α—tumor necrosis factor alpha; CHX—cycloheximide; ↑—enhancement.

Flavonoid	Cytotoxicity Model	Cytoprotective Pathway Induced	Reference
Baicalein	Oxidative stress	Iron chelation, ROS scavenging, Inhibition of complex I of ETC	[114] [132,133]
	I/R	Inhibition of apoptosis (Bcl-2 family proteins)	[148]
	Parkinson’s disease model	Mitogenesis	[174]
Catechins	Oxidative stress	↑ Antioxidant enzyme transcription Inhibition of complex I of ETC	[118,119] [136,137]
	Arsenic	Inhibition of apoptosis (Bcl-2 family proteins)	[156]
Kaempferol	Oxidative stress	Inhibition of complex I of ETC	[136]
	Doxorubicin-induced cardiotoxicity	Inhibition of apoptosis (Bcl-2 family proteins)	[161]
	Acute rotenone toxicity	Mitophagy	[180]
	Ischemic stroke model	Suppression of fission	[194]
Luteolin	I/R	Inhibition of apoptosis (Bcl-2 family proteins)	[146]
	Oxidative stress	Inhibition of complex I of ETC	[136]
Naringenin	Ischemic stroke	Inhibition of apoptosis (Bcl-2 family proteins)	[152]
	I/R	Activation of mitochondrial potassium channels	[200,201,202,203]
	TNF-α/CHX	Activation of mitochondrial potassium channels	[204]
Quercetin	Oxidative stress	Direct ROS scavenging from complex III of ETC, ↑ Antioxidant enzyme transcription Inhibition of complex I of ETC Inhibition of apoptosis (Bcl-2 family proteins)	[117] [120] [136] [153]
	I/R injury	Inhibition of apoptosis (Bcl-2 family proteins)	[147,150]
	Osteoarthritis	Mitogenesis	[176]
	Traumatic brain injury	Mitogenesis	[178]
	Chronic ethanol treatment	Mitophagy	[181]
	Non-alcoholic fatty liver disease model	Mitophagy	[182]
	Acute hypobaric hypoxia	Mitogenesis, inhibition of fission	[188]

## References

[1] Nichols M., Townsend N., Scarborough P., Rayner M. (2014). Cardiovascular disease in Europe 2014: Epidemiological update. Eur. Heart J..

[2] Stone G., Selker H., Thiele H., Patel M., Udelson J., Ohman E., Maehara A., Eitel I., CB G., PL J. (2016). Relationship between infarct size and outcomes following primary PCI: Patient-level analysis from 10 randomized trials. J. Am. Coll. Cardiol..

[3] Nabel E., Braunwald E. (2012). A tale of coronary artery disease and myocardial infarction. N. Engl. J. Med..

[4] Fricker M., Tolkovsky A., Borutaite V., Coleman M., Brown G. (2018). Neuronal cell death. Physiol. Rev..

[5] Sosa-Ortiz A., Acosta-Castillo I., Prince M. (2012). Epidemiology of dementias and Alzheimer’s disease. Arch. Med. Res..

[6] Tysnes O., Storstein A. (2017). Epidemiology of Parkinson’s disease. J. Neural Transm..

[7] Nezu M., Suzuki N. (2020). Roles of Nrf2 in protecting the kidney from oxidative damage. Int. J. Mol. Sci..

[8] Vasileva L., Savova M., Amirova K., Dinkova-Kostova A., Georgiev M. (2020). Obesity and NRF2-mediated cytoprotection: Where is the missing link. Pharm. Res..

[9] Lopes J.E., Leite H., Konstantyner T. (2019). Selenium and selenoproteins: From endothelial cytoprotection to clinical outcomes. Transl. Res. J. Lab. Clin. Med..

[10] Hakiminia B., Goudarzi A., Moghaddas A. (2019). Has Vitamin E any shreds of evidence in cisplatin-induced toxicity. J. Biochem. Mol. Toxicol..

[11] Peterson J., Dwyer J., Jacques P., McCullough M. (2015). Improving the estimation of flavonoid intake for study of health outcomes. Nutr. Rev..

[12] Rees A., Dodd G.F., Spencer J. (2018). The effects of flavonoids on cardiovascular health: A review of human intervention trials and implications for cerebrovascular function. Nutrients.

[13] Bondonno N., Dalgaard F., Kyrø C., Murray K., Bondonno C., Lewis J., Croft K., Gislason G., Scalbert A., Cassidy A. (2019). Flavonoid intake is associated with lower mortality in the danish diet cancer and health cohort. Nat. Commun..

[14] Heijnen C.G., Haenen G.R., van Acker F.A., van der Vijgh W.J., Bast A. (2001). Flavonoids as peroxynitrite scavengers: The role of the hydroxyl groups. Toxicol Vitr..

[15] Chun O.K., Kim D.O., Lee C.Y. (2003). Superoxide radical scavenging activity of the major polyphenols in fresh plums. J. Agric. Food Chem.

[16] Mira L., Fernandez M.T., Santos M., Rocha R., Florencio M.H., Jennings K.R. (2002). Interactions of flavonoids with iron and copper ions: A mechanism for their antioxidant activity. Free Radic Res..

[17] Cheng I.F., Breen K. (2000). On the ability of four flavonoids, baicilein, luteolin, naringenin, and quercetin, to suppress the Fenton reaction of the iron-ATP complex. Biometals.

[18] Williams R.J., Spencer J.P., Rice-Evans C. (2004). Flavonoids: Antioxidants or signalling molecules. Free Radic Biol. Med..

[19] Lee S.G., Kim B., Yang Y., Pham T.X., Park Y.K., Manatou J., Koo S.I., Chun O.K., Lee J.Y. (2014). Berry anthocyanins suppress the expression and secretion of proinflammatory mediators in macrophages by inhibiting nuclear translocation of NF-kappaB independent of NRF2-mediated mechanism. J. Nutr Biochem..

[20] Edirisinghe I., Banaszewski K., Cappozzo J., McCarthy D., Burton-Freeman B.M. (2011). Effect of black currant anthocyanins on the activation of endothelial nitric oxide synthase (eNOS) in vitro in human endothelial cells. J. Agric. Food Chem..

[21] Babu P.V., Liu D., Gilbert E.R. (2013). Recent advances in understanding the anti-diabetic actions of dietary flavonoids. J. Nutr. Biochem..

[22] Suh Y., Afaq F., Johnson J.J., Mukhtar H. (2009). A plant flavonoid fisetin induces apoptosis in colon cancer cells by inhibition of COX2 and Wnt/EGFR/NF-kappaB-signaling pathways. Carcinogenesis.

[23] Vauzour D., Vafeiadou K., Rodriguez-Mateos A., Rendeiro C., Spencer J.P. (2008). The neuroprotective potential of flavonoids: A multiplicity of effects. Genes Nutr..

[24] De Andrade Teles R., Diniz T., Costa Pinto T., De Oliveira Júnior R., Gama E., Silva M., De Lavor É., Fernandes A., De Oliveira A., De Almeida Ribeiro F. (2018). Flavonoids as therapeutic agents in Alzheimer’s and Parkinson’s diseases: A systematic review of preclinical evidences. Oxidative Med. Cell. Longev..

[25] Spagnuolo C., Moccia S., Russo G. (2018). Anti-inflammatory effects of flavonoids in neurodegenerative disorders. Eur. J. Med. Chem..

[26] Testai L. (2015). Flavonoids and mitochondrial pharmacology: A new paradigm for cardioprotection. Life Sci..

[27] McCullough M., Peterson J., Patel R., Jacques P., Shah R., Dwyer J. (2012). Flavonoid intake and cardiovascular disease mortality in a prospective cohort of us adults. Am. J. Clin. Nutr..

[28] Wang X., Ouyang Y., Liu J., Zhao G. (2014). Flavonoid intake and risk of CVD: A systematic review and meta-analysis of prospective cohort studies. Br. J. Nutr..

[29] Raman G., Avendano E., Chen S., Wang J., Matson J., Gayer B., Novotny J., Cassidy A. (2019). Dietary intakes of flavan-3-ols and cardiometabolic health: Systematic review and meta-analysis of randomized trials and prospective cohort studies. Am. J. Clin. Nutr..

[30] Kimble R., Keane K., Lodge J., Howatson G. (2019). Dietary intake of anthocyanins and risk of cardiovascular disease: A systematic review and meta-analysis of prospective cohort studies. Crit. Rev. Food Sci. Nutr..

[31] Heiss C., Sansone R., Karimi H., Krabbe M., Schuler D., Rodriguez-Mateos A., Kraemer T., Cortese-Krott M., Kuhnle G., Spencer J. (2015). Impact of cocoa flavanol intake on age-dependent vascular stiffness in healthy men: A randomized, controlled, double-masked trial. Age.

[32] Grassi D., Desideri G., Necozione S., di Giosia P., Barnabei R., Allegaert L., Bernaert H., Ferri C. (2015). Cocoa consumption dose-dependently improves flow-mediated dilation and arterial stiffness decreasing blood pressure in healthy individuals. J. Hypertens..

[33] Hollman P., Geelen A., Kromhout D. (2010). Dietary flavonol intake may lower stroke risk in men and women. J. Nutr..

[34] Shishtar E., Rogers G., Blumberg J., Au R., Jacques P. (2020). Long-term dietary flavonoid intake and risk of alzheimer disease and related dementias in the framingham offspring cohort. Am. J. Clin. Nutr..

[35] Maher P. (2017). Protective effects of fisetin and other berry flavonoids in Parkinson’s disease. Food Funct..

[36] Gao X., Cassidy A., Schwarzschild M.A., Rimm E.B., Ascherio A. (2012). Habitual intake of dietary flavonoids and risk of Parkinson disease. Neurology.

[37] Burton-Freeman B., Brzeziński M., Park E., Sandhu A., Xiao D., Edirisinghe I. (2019). A Selective Role of Dietary Anthocyanins and Flavan-3-ols in Reducing the Risk of Type 2 Diabetes Mellitus: A review of recent evidence. Nutrients.

[38] Pei R., Liu X., Bolling B. (2020). Flavonoids and gut health. Curr. Opin. Biotechnol..

[39] Oteiza P., Fraga C., Mills D., Taft D. (2018). Flavonoids and the gastrointestinal tract: Local and systemic effects. Mol. Asp. Med..

[40] Marais J., Deavours B., Dixon R., Fereira D., Grotewold E. (2006). The Stereochemistry of Flavonoids.

[41] Kumar S., Pandey A.K. (2013). Chemistry and biological activities of flavonoids: An overview. Sci. World J..

[42] Scalbert A. (1991). Antimicrobial properties of tannins. Phytochemistry.

[43] Ryan K.G., Swinny E.E., Markham K.R., Winefield C. (2002). Flavonoid gene expression and UV photoprotection in transgenic and mutant Petunia leaves. Phytochemistry.

[44] Xiao L., Carrillo J., Siemann E., Ding J. (2019). Herbivore-specific induction of indirect and direct defensive responses in leaves and roots. Aob Plants.

[45] Manach C., Scalbert A., Morand C., Remesy C., Jimenez L. (2004). Polyphenols: Food sources and bioavailability. Am. J. Clin. Nutr..

[46] Xiao J., Kai G., Yamamoto K., Chen X. (2013). Advance in dietary polyphenols as alpha-glucosidases inhibitors: A review on structure-activity relationship aspect. Crit. Rev. Food Sci. Nutr..

[47] Bhagwat S., Haytowitz D.B., Holden J.M. (2014). USDA Database for the Flavonoid Content of Selected Foods, Release 3.1.

[48] Arai Y., Watanabe S., Kimira M., Shimoi K., Mochizuki R., Kinae N. (2000). Dietary intakes of flavonols, flavones and isoflavones by Japanese women and the inverse correlation between quercetin intake and plasma LDL cholesterol concentration. J. Nutr..

[49] Rothwell J., Perez-Jimenez J., Neveu V., Medina-Remón A., M’hiri N., García-Lobato P., Manach C., Knox C., Eisner R., Wishart D. (2013). Phenol-explorer 3.0: A major update of the phenol-explorer database to incorporate data on the effects of food processing on polyphenol content. Database J. Biol. Databases Curation.

[50] Cassidy A., Minihane A. (2017). The role of metabolism (and the microbiome) in defining the clinical efficacy of dietary flavonoids. Am. J. Clin. Nutr..

[51] Murota K., Nakamura Y., Uehara M. (2018). Flavonoid metabolism: The interaction of metabolites and gut microbiota. Biosci. Biotechnol. Biochem..

[52] Nunnari J., Suomalainen A. (2012). Mitochondria: In sickness and in health. Cell.

[53] Spinelli J.B., Haigis M.C. (2018). The multifaceted contributions of mitochondria to cellular metabolism. Nat. Cell Biol..

[54] Tait S.W., Green D.R. (2012). Mitochondria and cell signalling. J. Cell Sci..

[55] Anderson A.J., Jackson T.D., Stroud D.A., Stojanovski D. (2019). Mitochondria-hubs for regulating cellular biochemistry: Emerging concepts and networks. Open Biol..

[56] Giorgi C., Marchi S., Pinton P. (2018). The machineries, regulation and cellular functions of mitochondrial calcium. Nat. Rev. Mol. Cell Biol..

[57] David G., Barrett E.F. (2003). Mitochondrial Ca^2+^ uptake prevents desynchronization of quantal release and minimizes depletion during repetitive stimulation of mouse motor nerve terminals. J. Physiol..

[58] Wiederkehr A., Szanda G., Akhmedov D., Mataki C., Heizmann C.W., Schoonjans K., Pozzan T., Spat A., Wollheim C.B. (2011). Mitochondrial matrix calcium is an activating signal for hormone secretion. Cell Metab..

[59] Antony A.N., Paillard M., Moffat C., Juskeviciute E., Correnti J., Bolon B., Rubin E., Csordas G., Seifert E.L., Hoek J.B. (2016). MICU1 regulation of mitochondrial Ca(2+) uptake dictates survival and tissue regeneration. Nat. Commun..

[60] Cheng J., Liao Y., Zhou L., Peng S., Chen H., Yuan Z. (2016). Amplified RLR signaling activation through an interferon-stimulated gene-endoplasmic reticulum stress-mitochondrial calcium uniporter protein loop. Sci. Rep..

[61] Collins Y., Chouchani E.T., James A.M., Menger K.E., Cocheme H.M., Murphy M.P. (2012). Mitochondrial redox signalling at a glance. J. Cell Sci..

[62] Figueira T., Barros M., Camargo A., Castilho R., Ferreira J., Kowaltowski A., Sluse F., Souza-Pinto N., Vercesi A. (2013). Mitochondria as a source of reactive oxygen and nitrogen species: From molecular mechanisms to human health. Antioxid. Redox Signal..

[63] Brand M. (2016). Mitochondrial generation of superoxide and hydrogen peroxide as the source of mitochondrial redox signaling. Free Radic. Biol. Med..

[64] West A.P., Brodsky I.E., Rahner C., Woo D.K., Erdjument-Bromage H., Tempst P., Walsh M.C., Choi Y., Shadel G.S., Ghosh S. (2011). TLR signalling augments macrophage bactericidal activity through mitochondrial ROS. Nature.

[65] Dai D.F., Johnson S.C., Villarin J.J., Chin M.T., Nieves-Cintron M., Chen T., Marcinek D.J., Dorn G.W., Kang Y.J., Prolla T.A. (2011). Mitochondrial oxidative stress mediates angiotensin II-induced cardiac hypertrophy and Galphaq overexpression-induced heart failure. Circ. Res..

[66] Leloup C., Tourrel-Cuzin C., Magnan C., Karaca M., Castel J., Carneiro L., Colombani A.L., Ktorza A., Casteilla L., Penicaud L. (2009). Mitochondrial reactive oxygen species are obligatory signals for glucose-induced insulin secretion. Diabetes.

[67] Sanjuan-Pla A., Cervera A.M., Apostolova N., Garcia-Bou R., Victor V.M., Murphy M.P., McCreath K.J. (2005). A targeted antioxidant reveals the importance of mitochondrial reactive oxygen species in the hypoxic signaling of HIF-1alpha. FEBS Lett..

[68] Olsen R.K., Cornelius N., Gregersen N. (2015). Redox signalling and mitochondrial stress responses; lessons from inborn errors of metabolism. J. Inherit. Metab Dis..

[69] Peoples J., Saraf A., Ghazal N., Pham T., Kwong J. (2019). Mitochondrial dysfunction and oxidative stress in heart disease. Exp. Mol. Med..

[70] Oberst A., Bender C., Green D.R. (2008). Living with death: The evolution of the mitochondrial pathway of apoptosis in animals. Cell Death Differ..

[71] Wei M.C., Zong W.X., Cheng E.H., Lindsten T., Panoutsakopoulou V., Ross A.J., Roth K.A., MacGregor G.R., Thompson C.B., Korsmeyer S.J. (2001). Proapoptotic BAX and BAK: A requisite gateway to mitochondrial dysfunction and death. Science.

[72] Del Re D., Amgalan D., Linkermann A., Liu Q., Kitsis R. (2019). Fundamental mechanisms of regulated cell death and implications for heart disease. Physiol. Rev..

[73] Tait S.W., Green D.R. (2013). Mitochondrial regulation of cell death. Cold Spring Harb Perspect Biol..

[74] Vaseva A.V., Marchenko N.D., Ji K., Tsirka S.E., Holzmann S., Moll U.M. (2012). p53 opens the mitochondrial permeability transition pore to trigger necrosis. Cell.

[75] Gao M., Yi J., Zhu J., Minikes A.M., Monian P., Thompson C.B., Jiang X. (2019). Role of mitochondria in ferroptosis. Mol. Cell.

[76] Fatokun A.A., Dawson V.L., Dawson T.M. (2014). Parthanatos: Mitochondrial-linked mechanisms and therapeutic opportunities. Br. J. Pharm..

[77] Nakagawa T., Shimizu S., Watanabe T., Yamaguchi O., Otsu K., Yamagata H., Inohara H., Kubo T., Tsujimoto Y. (2005). Cyclophilin _D_-dependent mitochondrial permeability transition regulates some necrotic but not apoptotic cell death. Nature.

[78] Becker T., Wagner R. (2018). Mitochondrial outer membrane channels: Emerging diversity in transport processes. Bioessays News Rev. Mol. Cell. Dev. Biol..

[79] Palmieri F., Pierri C. (2010). Mitochondrial metabolite transport. Essays Biochem..

[80] Smith C., Nehrke K., Brookes P. (2017). The slo(w) path to identifying the mitochondrial channels responsible for ischemic protection. Biochem. J..

[81] Szewczyk A., Bednarczyk P., Jędraszko J., Kampa R., Koprowski P., Krajewska M., Kucman S., Kulawiak B., Laskowski M., Rotko D. (2018). Mitochondrial potassium channels—an overview. Postepy Biochem..

[82] Gururaja Rao S., Ponnalagu D., Patel N., Singh H. (2018). Three decades of chloride intracellular channel proteins: From organelle to organ physiology. Curr. Protoc. Pharm..

[83] Bachmann M., Pontarin G., Szabo I. (2019). The Contribution of mitochondrial ion channels to cancer development and progression. Cell. Physiol. Biochem. Int. J. Exp. Cell. Physiol. Biochem. Pharm..

[84] Checchetto V., Azzolini M., Peruzzo R., Capitanio P., Leanza L. (2018). Mitochondrial potassium channels in cell death. Biochem. Biophys. Res. Commun..

[85] Krabbendam I., Honrath B., Culmsee C., Dolga A. (2018). Mitochondrial Ca^2+^-activated K^+^ channels and their role in cell life and death pathways. Cell Calcium.

[86] Szabo I., Zoratti M. (2014). Mitochondrial channels: Ion fluxes and more. Physiol Rev..

[87] Garlid K., Paucek P., Yarov-Yarovoy V., Murray H., Darbenzio R., D’Alonzo A., Lodge N., Smith M., Grover G. (1997). Cardioprotective effect of diazoxide and its interaction with mitochondrial ATP-sensitive K^+^ channels. Possible mechanism of cardioprotection. Circ. Res..

[88] Gross G., Fryer R. (1999). Sarcolemmal Versus Mitochondrial ATP-sensitive K+ channels and myocardial preconditioning. Circ. Res..

[89] Laskowski M., Augustynek B., Kulawiak B., Koprowski P., Bednarczyk P., Jarmuszkiewicz W., Szewczyk A. (2016). What do we not know about mitochondrial potassium channels?. Biochim Biophys Acta.

[90] Murry C., Jennings R., Reimer K. (1986). Preconditioning with ischemia: A delay of lethal cell injury in ischemic myocardium. Circulation.

[91] Liu Y., Sato T., O’Rourke B., Marban E. (1998). Mitochondrial ATP-dependent potassium channels: Novel effectors of cardioprotection. Circulation.

[92] Wang L., Zhu Q., Wang G., Deng T., Chen R., Liu M., Wang S. (2011). The protective roles of mitochondrial ATP-sensitive potassium channels during hypoxia-ischemia-reperfusion in brain. Neurosci. Lett..

[93] Grover G., Burkett D., Parham C., Scalese R., Sadanaga K. (2003). Protective effect of mitochondrial KATP activation in an isolated gracilis model of ischemia and reperfusion in dogs. J. Cardiovasc. Pharm..

[94] Ohya S., Kuwata Y., Sakamoto K., Muraki K., Imaizumi Y. (2005). Cardioprotective effects of estradiol include the activation of large-conductance Ca(2+)-activated K(+) channels in cardiac mitochondria. Am. J. Physiol. Heart Circ. Physiol..

[95] Xu W., Liu Y., Wang S., McDonald T., Van Eyk J.E., Sidor A., O’Rourke B. (2002). Cytoprotective role of Ca^2+^- activated K^+^ channels in the cardiac inner mitochondrial membrane. Science.

[96] Szewczyk A., Marbán E. (1999). Mitochondria: A new target for K channel openers. Trends Pharm. Sci..

[97] Borchert G., Yang C., Kolár F. (2011). Mitochondrial BKCa channels contribute to protection of cardiomyocytes isolated from chronically hypoxic rats. Am. J. Physiol. Heart Circ. Physiol..

[98] Yan X., Guo X., Jiao F., Liu X., Liu Y. (2015). Activation of large-conductance Ca(2+)-activated K(+) channels inhibits glutamate-induced oxidative stress through attenuating ER stress and mitochondrial dysfunction. Neurochem. Int..

[99] Heinen A., Aldakkak M., Stowe D., Rhodes S., Riess M., Varadarajan S., Camara A. (2007). Reverse electron flow-induced ROS production is attenuated by activation of mitochondrial Ca^2+^-sensitive K^+^ channels. Am. J. Physiol. Heart. Circ. Physiol..

[100] Facundo H., De Paula J., Kowaltowski A. (2007). Mitochondrial ATP-sensitive K^+^ channels are redox-sensitive pathways that control reactive oxygen species production. Free Radic. Biol. Med..

[101] Kulawiak B., Kudin A., Szewczyk A., Kunz W. (2008). BK Channel openers inhibit ROS production of isolated rat brain mitochondria. Exp. Neurol..

[102] Chouchani E., Pell V., Gaude E., Aksentijević D., Sundier S., Robb E., Logan A., Nadtochiy S., Ord E., Smith A. (2014). Ischaemic accumulation of succinate controls reperfusion injury through mitochondrial ROS. Nature.

[103] Murata M., Akao M., O’Rourke B., Marbán E. (2001). Mitochondrial ATP-sensitive potassium channels attenuate matrix Ca(2+) overload during simulated ischemia and reperfusion: Possible mechanism of cardioprotection. Circ. Res..

[104] Facundo H., Fornazari M., Kowaltowski A. (2006). Tissue protection mediated by mitochondrial K^+^ channels. Biochim. Et Biophys. Acta.

[105] Sastre J., Pallardo F.V., Vina J. (2000). Mitochondrial oxidative stress plays a key role in aging and apoptosis. Iubmb Life.

[106] Takabe W., Niki E., Uchida K., Yamada S., Satoh K., Noguchi N. (2001). Oxidative stress promotes the development of transformation: Involvement of a potent mutagenic lipid peroxidation product, acrolein. Carcinogenesis.

[107] Kawanishi S., Hiraku Y., Oikawa S. (2001). Mechanism of guanine-specific DNA damage by oxidative stress and its role in carcinogenesis and aging. Mutat Res..

[108] Gracy R.W., Talent J.M., Kong Y., Conrad C.C. (1999). Reactive oxygen species: The unavoidable environmental insult?. Mutat Res..

[109] Di Meo S., Reed T.T., Venditti P., Victor V.M. (2016). Role of ROS and RNS sources in physiological and pathological conditions. Oxid Med. Cell Longev..

[110] Sichel G., Corsaro C., Scalia M., Di Bilio A.J., Bonomo R.P. (1991). In vitro scavenger activity of some flavonoids and melanins against O_2_^−dot^. Free Radic Biol Med..

[111] Cao G., Sofic E., Prior R.L. (1997). Antioxidant and prooxidant behavior of flavonoids: Structure-activity relationships. Free Radic Biol Med..

[112] Fraga C. (2007). Plant polyphenols: How to translate their in vitro antioxidant actions to in vivo conditions. Iubmb Life.

[113] Miyagi Y., Miwa K., Inoue H. (1997). Inhibition of human low-density lipoprotein oxidation by flavonoids in red wine and grape juice. Am. J. Cardiol..

[114] Perez C.A., Wei Y., Guo M. (2009). Iron-binding and anti-Fenton properties of baicalein and baicalin. J. Inorg Biochem..

[115] Bastianetto S., Quirion R. (2002). Natural extracts as possible protective agents of brain aging. Neurobiol Aging.

[116] Esselun C., Bruns B., Hagl S., Grewal R., Eckert G. (2019). Differential effects of silibinin a on mitochondrial function in neuronal PC12 and HepG2 liver cells. Oxidative Med. Cell. Longev..

[117] Dudylina A., Ivanova M., Shumaev K., Ruuge E. (2019). Superoxide formation in cardiac mitochondria and effect of phenolic antioxidants. Cell Biochem. Biophys..

[118] Shah Z.A., Li R., Ahmad A.S., Kensler T.W., Yamamoto M., Biswal S., Doré S. (2010). The flavanol (−)-epicatechin prevents stroke damage through the Nrf2/HO1 pathway. J. Cereb Blood Flow Metab..

[119] Assuncao M., Santos-Marques M.J., Carvalho F., Andrade J.P. (2010). Green tea averts age-dependent decline of hippocampal signaling systems related to antioxidant defenses and survival. Free Radic. Biol. Med..

[120] Arredondo F., Echeverry C., Abin-Carriquiry J., Blasina F., Antúnez K., Jones D., Go Y., Liang Y., Dajas F. (2010). After Cellular internalization, quercetin causes Nrf2 nuclear translocation, increases Glutathione levels, and prevents neuronal death against an oxidative insult. Free Radic. Biol. Med..

[121] Schaffer S., Asseburg H., Kuntz S., Muller W., Eckert G. (2012). Effects of polyphenols on brain ageing and Alzheimer’s disease: Focus on mitochondria. Mol. Neurobiol..

[122] Alvarez-Suarez J., Giampieri F., Cordero M., Gasparrini M., Forbes-Hernández T., Mazzoni L., Afrin S., Beltrán-Ayala P., González-Paramás A., Santos-Buelga C. (2016). Activation of AMPK/Nrf2 signalling by Manuka honey protects human dermal fibroblasts against oxidative damage by improving antioxidant response and mitochondrial function promoting wound healing. J. Funct. Foods.

[123] Luo Y., Cui H.X., Jia A., Jia S.S., Yuan K. (2018). The Protective effect of the total flavonoids of abelmoschus esculentus l. Flowers on transient cerebral ischemia-reperfusion injury is due to activation of the Nrf2-ARE pathway. Oxid Med. Cell Longev..

[124] Dai C., Tang S., Wang Y., Velkov T., Xiao X. (2017). Baicalein acts as a nephroprotectant that ameliorates colistin-induced nephrotoxicity by activating the antioxidant defence mechanism of the kidneys and down-regulating the inflammatory response. J. Antimicrob. Chemother..

[125] Arulselvan P., Fard M., Tan W., Gothai S., Fakurazi S., Norhaizan M., Kumar S. (2016). Role of antioxidants and natural products in inflammation. Oxidative Med. Cell. Longev..

[126] Zhou Z., Xie S., Saw W., Ho P., Wang H., Lei Z., Yi Z., Tan E. (2019). The therapeutic implications of tea polyphenols against dopamine (DA) neuron degeneration in Parkinson’s disease (PD). Cells.

[127] Heim K.E., Tagliaferro A.R., Bobilya D.J. (2002). Flavonoid antioxidants: Chemistry, metabolism and structure-activity relationships. J. Nutr Biochem..

[128] Sandoval-Acuña C., Ferreira J., Speisky H. (2014). Polyphenols and mitochondria: An update on their increasingly emerging ROS-scavenging independent actions. Arch. Biochem. Biophys..

[129] McAnlis G., McEneny J., Pearce J., Young I. (1999). Absorption and antioxidant effects of quercetin from onions, in man. Eur. J. Clin. Nutr..

[130] Cheng G., Zielonka J., McAllister D., Hardy M., Ouari O., Joseph J., Dwinell M., Kalyanaraman B. (2015). Antiproliferative effects of mitochondria-targeted cationic antioxidants and analogs: Role of mitochondrial bioenergetics and energy-sensing mechanism. Cancer Lett..

[131] Brown J.E., Khodr H., Hider R.C., Rice-Evans C.A. (1998). Structural dependence of flavonoid interactions with Cu2+ ions: Implications for their antioxidant properties. Biochem, J..

[132] Hodnick W., Bohmont C., Capps C., Pardini R. (1987). Inhibition of the mitochondrial NADH-oxidase (NADH-coenzyme Q oxido-reductase) enzyme system by flavonoids: A structure-activity study. Biochem. Pharm..

[133] Hodnick W., Duval D., Pardini R. (1994). Inhibition of mitochondrial respiration and cyanide-stimulated generation of reactive oxygen species by selected flavonoids. Biochem. Pharm..

[134] Dabaghi-Barbosa P., Mariante Rocha A., Franco da Cruz Lima A., Heleno de Oliveira B., Benigna Martinelli de Oliveira M., Gunilla Skare Carnieri E., Cadena S., Eliane Merlin Rocha M. (2005). Hispidulin: Antioxidant properties and effect on mitochondrial energy metabolism. Free Radic. Res..

[135] Herrerias T., de Oliveira B., Gomes M., de Oliveira M., Carnieri E., Cadena S., Martinez G., Rocha M. (2008). Eupafolin: Effect on mitochondrial energetic metabolism. Bioorganic Med. Chem..

[136] Lagoa R., Graziani I., Lopez-Sanchez C., Garcia-Martinez V., Gutierrez-Merino C. (2011). Complex I and cytochrome c are molecular targets of flavonoids that inhibit hydrogen peroxide production by mitochondria. Biochim. Et Biophys. Acta.

[137] Iglesias D., Bombicino S., Boveris A., Valdez L. (2019). (+)-catechin inhibits heart mitochondrial complex i and nitric oxide synthase: Functional consequences on membrane potential and hydrogen peroxide production. Food Funct..

[138] Sharikadze N., Jojua N., Sepashvili M., Zhuravliova E., Mikeladze D. (2016). Mitochondrial target of nobiletin’s action. Nat. Prod. Commun..

[139] Carrasco-Pozo C., Gotteland M., Speisky H. (2011). Apple peel polyphenol extract protects against indomethacin-induced damage in Caco-2 cells by preventing mitochondrial complex i inhibition. J. Agric. Food Chem..

[140] Dorta D., Pigoso A., Mingatto F., Rodrigues T., Prado I., Helena A., Uyemura S., Santos A., Curti C. (2005). The interaction of flavonoids with mitochondria: Effects on energetic processes. Chem. -Biol. Interact..

[141] Dhiman P., Malik N., Sobarzo-Sánchez E., Uriarte E., Khatkar A. (2019). Quercetin and related chromenone derivatives as monoamine oxidase inhibitors: Targeting neurological and mental disorders. Molecules.

[142] Kashyap D., Garg V., Tuli H., Yerer M., Sak K., Sharma A., Kumar M., Aggarwal V., Sandhu S. (2019). Fisetin and quercetin: Promising flavonoids with chemopreventive potential. Biomolecules.

[143] Kopustinskiene D.M., Jakstas V., Savickas A., Bernatoniene J. (2020). Flavonoids as anticancer agents. Nutrients.

[144] Naoi M., Wu Y., Shamoto-Nagai M., Maruyama W. (2019). Mitochondria in neuroprotection by phytochemicals: Bioactive polyphenols modulate mitochondrial apoptosis system, function and structure. Int. J. Mol. Sci..

[145] Zeng C., Jiang W., Zheng R., He C., Li J., Xing J. (2018). Cardioprotection of tilianin ameliorates myocardial ischemia-reperfusion injury: Role of the apoptotic signaling pathway. PLoS ONE.

[146] Fang F., Li D., Pan H., Chen D., Qi L., Zhang R., Sun H. (2011). Luteolin inhibits apoptosis and improves cardiomyocyte contractile function through the PI3K/Akt pathway in simulated ischemia/reperfusion. Pharmacology.

[147] Tinay I., Sener T., Cevik O., Cadirci S., Toklu H., Cetinel S., Sener G., Tarcan T. (2017). Antioxidant agent quercetin prevents impairment of bladder tissue contractility and apoptosis in a rat model of ischemia/reperfusion injury. Low. Urin. Tract Symptoms.

[148] Lai C., Huang P., Yang A., Chiang S., Tang C., Tseng K., Huang C. (2017). Baicalein attenuates lung injury induced by myocardial ischemia and reperfusion. Am. J. Chin. Med..

[149] Jian J., Xuan F., Qin F., Huang R. (2016). The antioxidant, anti-inflammatory and anti-apoptotic activities of the *Bauhinia championii* flavone are connected with protection against myocardial ischemia/reperfusion injury. Cell. Physiol. Biochem. Int. J. Exp. Cell. Physiol. Biochem. Pharm..

[150] Yang T., Kong B., Gu J., Kuang Y., Cheng L., Yang W., Xia X., Shu H. (2014). Anti-apoptotic and anti-oxidative roles of quercetin after traumatic brain injury. Cell. Mol. Neurobiol..

[151] Han Y., Zhang T., Su J., Zhao Y., Chenchen N., Wang N., Li X. (2017). Apigenin attenuates oxidative stress and neuronal apoptosis in early brain injury following subarachnoid hemorrhage. J. Clin. Neurosci..

[152] Wang K., Chen Z., Huang J., Huang L., Luo N., Liang X., Liang M., Xie W. (2017). Naringenin prevents ischaemic stroke damage via anti-apoptotic and anti-oxidant effects. Clin. Exp. Pharm. Physiol..

[153] Bournival J., Quessy P., Martinoli M. (2009). Protective effects of resveratrol and quercetin against MPP^+^-induced oxidative stress act by modulating markers of apoptotic death in dopaminergic neurons. Cell. Mol. Neurobiol..

[154] Guo B., Zheng C., Cai W., Cheng J., Wang H., Li H., Sun Y., Cui W., Wang Y., Han Y. (2016). Multifunction of chrysin in parkinson’s model: Anti-neuronal apoptosis, neuroprotection via activation of MEF2D, and inhibition of monoamine oxidase-B. J. Agric. Food Chem..

[155] Wang D., Li S., Zhu X., Wang Y., Wu W., Zhang X. (2013). Protective effects of hesperidin against amyloid-β (Aβ) induced neurotoxicity through the voltage dependent anion channel 1 (VDAC1)-mediated mitochondrial apoptotic pathway in PC12 cells. Neurochem. Res..

[156] Yu N., Pei H., Huang Y., Li Y. (2017). (−)-Epigallocatechin-3-gallate inhibits arsenic-induced inflammation and apoptosis through suppression of oxidative stress in mice. Cell. Physiol. Biochem. Int. J. Exp. Cell. Physiol. Biochem. Pharm..

[157] Yang Y., Gong X., Huang L., Wang Z., Wan R., Zhang P., Zhang Q., Chen Z., Zhang B. (2017). Diosmetin exerts anti-oxidative, anti-inflammatory and anti-apoptotic effects to protect against endotoxin-induced acute hepatic failure in mice. Oncotarget.

[158] Arjinajarn P., Chueakula N., Pongchaidecha A., Jaikumkao K., Chatsudthipong V., Mahatheeranont S., Norkaew O., Chattipakorn N., Lungkaphin A. (2017). Anthocyanin-rich riceberry bran extract attenuates gentamicin-induced hepatotoxicity by reducing oxidative stress, inflammation and apoptosis in rats. Biomed. Pharmacother..

[159] Zare M., Rakhshan K., Aboutaleb N., Nikbakht F., Naderi N., Bakhshesh M., Azizi Y. (2019). Apigenin attenuates doxorubicin induced cardiotoxicity via reducing oxidative stress and apoptosis in male rats. Life Sci..

[160] Malik S., Bhatia J., Suchal K., Gamad N., Dinda A., Gupta Y., Arya D. (2015). Nobiletin ameliorates cisplatin-induced acute kidney injury due to its anti-oxidant, anti-inflammatory and anti-apoptotic effects. Exp. Toxicol. Pathol. Off. J. Ges. Fur Toxikol. Pathol..

[161] Xiao J., Sun G., Sun B., Wu Y., He L., Wang X., Chen R., Cao L., Ren X., Sun X. (2012). Kaempferol protects against doxorubicin-induced cardiotoxicity in vivo and in vitro. Toxicology.

[162] Ploumi C., Daskalaki I., Tavernarakis N. (2017). Mitochondrial biogenesis and clearance: A balancing act. FEBS J..

[163] Simmons E., Scholpa N., Schnellmann R. (2020). Mitochondrial biogenesis as a therapeutic target for traumatic and neurodegenerative CNS diseases. Exp. Neurol..

[164] Shao D., Liu Y., Liu X., Zhu L., Cui Y., Cui A.A.Q., Kong X., Liu Y., Chen Q. (2010). PGC-1 Beta-regulated mitochondrial biogenesis and function in myotubes is mediated by NRF-1 and ERR alpha. Mitochondrion.

[165] Salma N., Song J., Arany Z., Fisher D. (2015). Transcription factor Tfe3 directly regulates Pgc-1alpha in muscle. J. Cell. Physiol..

[166] Rasbach K., Schnellmann R. (2008). Isoflavones promote mitochondrial biogenesis. J. Pharm. Exp..

[167] Davis J., Murphy E., Carmichael M., Davis B. (2009). Quercetin increases brain and muscle mitochondrial biogenesis and exercise tolerance. Am. J. Physiol. Regul. Integr. Comp. Physiol..

[168] Nieman D., Williams A., Shanely R., Jin F., McAnulty S., Triplett N., Austin M., Henson D. (2010). Quercetin’s influence on exercise performance and muscle mitochondrial biogenesis. Med. Sci. Sports Exerc..

[169] Rayamajhi N., Kim S., Go H., Joe Y., Callaway Z., Kang J., Ryter S., Chung H. (2013). Quercetin induces mitochondrial biogenesis through activation of HO-1 in HepG2 cells. Oxidative Med. Cell. Longev..

[170] Yoshino M., Naka A., Sakamoto Y., Shibasaki A., Toh M., Tsukamoto S., Kondo K., Iida K. (2015). Dietary isoflavone daidzein promotes tfam expression that increases mitochondrial biogenesis in C_2_C_12_ muscle cells. J. Nutr. Biochem..

[171] Jung H., Lee D., Ryu H., Choi B., Go Y., Lee N., Lee D., Son H., Jeon J., Kim S. (2017). Myricetin improves endurance capacity and mitochondrial density by activating SIRT1 and PGC-1α. Sci. Rep..

[172] Lee M., Kim Y. (2018). Effects of isorhamnetin on adipocyte mitochondrial biogenesis and AMPK activation. Molecules.

[173] Kou G., Li Z., Wu C., Liu Y., Hu Y., Guo L., Xu X., Zhou Z. (2018). Citrus tangeretin improves skeletal muscle mitochondrial biogenesis via activating the AMPK-PGC1-α pathway in vitro and in vivo: A possible mechanism for its beneficial effect on physical performance. J. Agric. Food Chem..

[174] Zhang X., Du L., Zhang W., Yang Y., Zhou Q., Du G. (2017). Therapeutic effects of baicalein on rotenone-induced Parkinson’s disease through protecting mitochondrial function and biogenesis. Sci. Rep..

[175] Chen X., Wang L., Wu Y., Song S., Min H., Yang Y., He X., Liang Q., Yi L., Wang Y. (2018). Effect of puerarin in promoting fatty acid oxidation by increasing mitochondrial oxidative capacity and biogenesis in skeletal muscle in diabetic rats. Nutr. Diabetes.

[176] Qiu L., Luo Y., Chen X. (2018). Quercetin attenuates mitochondrial dysfunction and biogenesis via upregulated AMPK/SIRT1 signaling pathway in OA rats. Biomed. Pharmacother..

[177] Wei L., Sun X., Qi X., Zhang Y., Li Y., Xu Y. (2019). Dihydromyricetin ameliorates cardiac ischemia/reperfusion injury through Sirt3 activation. Biomed. Res. Int..

[178] Li X., Wang H., Wen G., Li L., Gao Y., Zhuang Z., Zhou M., Mao L., Fan Y. (2018). Neuroprotection by quercetin via mitochondrial function adaptation in traumatic brain injury: PGC-1α pathway as a potential mechanism. J. Cell. Mol. Med..

[179] Youle R., Narendra D. (2011). Mechanisms of mitophagy. Nat. Rev. Mol. Cell Biol..

[180] Filomeni G., Graziani I., De Zio D., Dini L., Centonze D., Rotilio G., Ciriolo M. (2012). Neuroprotection of kaempferol by autophagy in models of rotenone-mediated acute toxicity: Possible implications for Parkinson’s disease. Neurobiol. Aging.

[181] Yu X., Xu Y., Zhang S., Sun J., Liu P., Xiao L., Tang Y., Liu L., Yao P. (2016). Quercetin attenuates chronic ethanol-induced hepatic mitochondrial damage through enhanced mitophagy. Nutrients.

[182] Liu P., Lin H., Xu Y., Zhou F., Wang J., Liu J., Zhu X., Guo X., Tang Y., Yao P. (2018). Frataxin-mediated PINK1-parkin-dependent mitophagy in hepatic steatosis: The protective effects of quercetin. Mol. Nutr. Food Res..

[183] Chen X., Yi L., Song S., Wang L., Liang Q., Wang Y., Wu Y., Gao Q. (2018). Puerarin attenuates palmitate-induced mitochondrial dysfunction, impaired mitophagy and inflammation in L6 myotubes. Life Sci..

[184] Feng J., Chen X., Lu S., Li W., Yang D., Su W., Wang X., Shen J. (2018). Naringin attenuates cerebral ischemia-reperfusion injury through inhibiting peroxynitrite-mediated mitophagy activation. Mol. Neurobiol..

[185] Tilokani L., Nagashima S., Paupe V., Prudent J. (2018). Mitochondrial dynamics: Overview of molecular mechanisms. Essays Biochem..

[186] Kraus F., Ryan M. (2017). The constriction and scission machineries involved in mitochondrial fission. J. Cell Sci..

[187] Pernas L., Scorrano L. (2016). Mito-morphosis: Mitochondrial fusion, fission, and cristae remodeling as key mediators of cellular function. Annu. Rev. Physiol..

[188] Liu P., Zou D., Yi L., Chen M., Gao Y., Zhou R., Zhang Q., Zhou Y., Zhu J., Chen K. (2015). Quercetin ameliorates hypobaric hypoxia-induced memory impairment through mitochondrial and neuron function adaptation via the PGC-1α pathway. Restor. Neurol. Neurosci..

[189] Cui L., Li Z., Chang X., Cong G., Hao L. (2017). Quercetin attenuates vascular calcification by inhibiting oxidative stress and mitochondrial fission. Vasc. Pharm..

[190] Chen C., Huang J., Shen J., Bai Q. (2019). Quercetin improves endothelial insulin sensitivity in obese mice by inhibiting drp1 phosphorylation at serine 616 and mitochondrial fragmentation. Acta Biochim. Et Biophys. Sin..

[191] Parrado-Fernández C., Sandebring-Matton A., Rodriguez-Rodriguez P., Aarsland D., Cedazo-Mínguez A. (2016). Anthocyanins protect from complex i inhibition and APPswe mutation through modulation of the mitochondrial fission/fusion pathways. Biochim. Et Biophys. Acta.

[192] Yang X., Liu T., Chen B., Wang F., Yang Q., Chen X. (2017). Grape seed proanthocyanidins prevent irradiation-induced differentiation of human lung fibroblasts by ameliorating mitochondrial dysfunction. Sci. Rep..

[193] Li S., Sun X., Xu L., Sun R., Ma Z., Deng X., Liu B., Fu Q., Qu R., Ma S. (2017). Baicalin attenuates in vivo and in vitro hyperglycemia-exacerbated ischemia/reperfusion injury by regulating mitochondrial function in a manner dependent on AMPK. Eur. J. Pharm..

[194] Wu B., Luo H., Zhou X., Cheng C., Lin L., Liu B., Liu K., Li P., Yang H. (2017). Succinate-induced neuronal mitochondrial fission and hexokinase ii malfunction in ischemic stroke: Therapeutical effects of kaempferol. Biochim. Et Biophys. Acta. Mol. Basis Dis..

[195] Huang Y., Chen K., Ren Q., Yi L., Zhu J., Zhang Q., Mi M. (2018). Dihydromyricetin attenuates dexamethasone-induced muscle atrophy by improving mitochondrial function via the PGC-1α pathway. Cell. Physiol. Biochem. Int. J. Exp. Cell. Physiol. Biochem. Pharm..

[196] Son E., Kim S., Ryter S., Yeo E., Kyung S., Kim Y., Jeong S., Lee C., Park J. (2018). Quercetogetin protects against cigarette smoke extract-induced apoptosis in epithelial cells by inhibiting mitophagy. Toxicol. Vitr..

[197] Gao Q., Pan H., Qiu S., Lu Y., Bruce I., Luo J., Xia Q. (2006). Atractyloside and 5-hydroxydecanoate block the protective effect of puerarin in isolated rat heart. Life Sci..

[198] Couvreur N., Tissier R., Pons S., Chenoune M., Waintraub X., Berdeaux A., Ghaleh B. (2009). The ceiling effect of pharmacological postconditioning with the phytoestrogen genistein is reversed by the gsk3beta inhibitor SB 216763 [3-(2,4-dichlorophenyl)-4(1-methyl-1H-indol-3-yl)-1H-pyrrole-2,5-dione] through mitochondrial ATP-dependent potassium channel opening. J. Pharm. Exp..

[199] Hu Y., Li L., Yin W., Shen L., You B., Gao H. (2014). Protective effect of proanthocyanidins on anoxia-reoxygenation injury of myocardial cells mediated by the PI3K/Akt/GSK-3β pathway and mitochondrial ATP-sensitive potassium channel. Mol. Med. Rep..

[200] Meng L., Ma H., Guo H., Kong Q., Zhang Y. (2016). The cardioprotective effect of naringenin against ischemia-reperfusion injury through activation of ATP-sensitive potassium channel in rat. Can. J. Physiol. Pharm..

[201] Testai L., Martelli A., Marino A., D’Antongiovanni V., Ciregia F., Giusti L., Lucacchini A., Chericoni S., Breschi M., Calderone V. (2013). The activation of mitochondrial BK potassium channels contributes to the protective effects of naringenin against myocardial ischemia/reperfusion injury. Biochem. Pharm..

[202] Testai L., Da Pozzo E., Piano I., Pistelli L., Gargini C., Breschi M., Braca A., Martini C., Martelli A., Calderone V. (2017). The citrus flavanone naringenin produces cardioprotective effects in hearts from 1 year old rat, through activation of mitoBK channels. Front. Pharm..

[203] Kampa R.P., Kicinska A., Jarmuszkiewicz W., Pasikowska-Piwko M., Dolegowska B., Debowska R., Szewczyk A., Bednarczyk P. (2019). Naringenin as an opener of mitochondrial potassium channels in dermal fibroblasts. Exp. Derm..

[204] Kicinska A., Kampa R., Daniluk J., Sek A., Jarmuszkiewicz W., Szewczyk A., Bednarczyk P. (2020). Regulation of the mitochondrial BKCa channel by the citrus flavonoid naringenin as a potential means of preventing cell damage. Molecules.

[205] Kyselova Z. (2011). Toxicological aspects of the use of phenolic compounds in disease prevention. Interdiscip Toxicol.

